# Neuroprotective Effects of Cistanches Herba Therapy on Patients with Moderate Alzheimer's Disease

**DOI:** 10.1155/2015/103985

**Published:** 2015-09-07

**Authors:** Nan Li, Jianping Wang, Jun Ma, Zhiqiang Gu, Chao Jiang, Lie Yu, Xiaojie Fu

**Affiliations:** ^1^Department of Neurology, The Fifth Affiliated Hospital of Zhengzhou University, Zhengzhou, Henan 450052, China; ^2^Department of Neurology, The Second Affiliated Hospital of Zhengzhou University, Zhengzhou, Henan 450014, China; ^3^Department of Gastroenterology, The Second Affiliated Hospital of Zhengzhou University, Zhengzhou, Henan 450052, China; ^4^Department of Radiology, The Second Affiliated Hospital of Zhengzhou University, Zhengzhou, Henan 450014, China

## Abstract

Cistanches Herba (CH) is thought to be a “Yang-invigorating” material in traditional Chinese medicine. We evaluated neuroprotective effects of Cistanches Herba on Alzheimer's disease (AD) patients. Moderate AD participants were divided into 3 groups: Cistanches Herba capsule (CH, *n* = 10), Donepezil tablet (DON, *n* = 8), and control group without treatment (*n* = 6). We assessed efficacy by MMSE and ADAS-cog, and investigated the volume changes of hippocampus by 1.5 T MRI scans. Protein, mRNA levels, and secretions of total-tau (T-tau), tumor necrosis factor-*α* (TNF-*α*), and interleukin- (IL) 1*β* (IL-1*β*) in cerebrospinal fluid (CSF) were detected by Western blot, RT-PCR, and ELISA. The scores showed statistical difference after 48 weeks of treatment compared to control group. Meanwhile, volume changes of hippocampus were slight in drug treatment groups but distinct in control group; the levels of T-tau, TNF-*α*, and IL-1*β* were decreased compared to those in control group. Cistanches Herba could improve cognitive and independent living ability of moderate AD patients, slow down volume changes of hippocampus, and reduce the levels of T-tau, TNF-*α*, and IL-1*β*. It suggested that Cistanches Herba had potential neuroprotective effects for moderate AD.

## 1. Introduction

Alzheimer's disease (AD) is a series of clinical syndrome caused by neurodegeneration that is characterized by progressive deterioration in cognitive ability and capacity for independent living. It is estimated that there are about 28.4 million people of AD worldwide in 2010 [1]. Now no disease-modifying therapies to prevent or treat AD are available. Hyperphosphorylated tau protein that is aggregating intracellularly in the AD brain and building neurofibrillary tangles and amyloid-beta (A*β*) protein that is accumulating extracellular in the AD brain and building cortical senile plaques are the main neuropathological marks of AD. Increased contents of total-tau (T-tau) in the cerebrospinal fluid (CSF) have been consistently demonstrated in AD [2]. The progressive atrophy of various brain regions in AD is also widely observed, particularly in the hippocampus which is a region involved in cognition, memory, and learning. Postmortem reports showed early neuropathological lesions and neuronal loss in hippocampus [3]. As magnetic resonance imaging (MRI) can structurally track hippocampus atrophy in the mid- and late-states of AD, it can be another marker of AD [4]. Recently neuroinflammation from abnormally activated neuroglia emerged as an important mechanism of AD process [5]. The increased levels of A*β* and the deposition cause chronic activation of the immune system and disorder of microglial clearance functions. The epidemiological data indicated a key role in activation of the native immune system as a disease-promoting factor [6]. The cytokines are believed to participate in the accumulation of amyloid plaques and accelerate the harmful effects of A*β* through involvement in inflammatory process [7]. Increasing evidences reveal that brain can initiate a well-regulated immune response to injury [8, 9] and motivate the peripheral immune cells [10, 11]. Circulating cytokines can cross the blood brain barrier (BBB) by saturable transport mechanism [12, 13]. TNF-*α*, IL-1*β*, and proinflammatory cytokines have already been proved to be induced in mice brains by ionizing radiation [14, 15]. In addition, IL-1*β* may have different effects on CNS cells: low concentration of IL-1*β* has protective effects on cultured cortical neurons against excitotoxicity, but high concentration of IL-1*β* has neurotoxic effects [16]. Numerous researches suggest a central role of proinflammatory cytokines in CNS inflammation [17–21], and the high levels of those cytokines may cause neuronal damage in AD brain [22]. Currently some anti-inflammatory therapeutic ideas of the treatment in AD have been proposed.

Cistanches Herba (CH), holoparasitic plant of* Cistanche deserticola* Y.C. Ma which is characterized as the major material to tonify the kidney and a “Yang-invigorating” material in traditional Chinese medicine theory [23]. It is widely used to treat forgetfulness and loss of hearing, increase the strength of reproduction, and develop fertility function. The previous studies have demonstrated that CH has different pharmacological activities: antinociceptive and anti-inflammatory effects [24–26]. The primary active compounds in CH are phenylethanoid glycosides: such as verbascoside and Echinacoside [27], which are thought to have some effects on center nervous system (CNS). Studies have reported that verbascoside shows neuroprotective effects against 1-methyl-4-phenylpyridium (MPP+) 5 which has been proved to induce memory deficits significantly in vivo [28]. Echinacoside can rescue SH-SY5Y neuronal cells following TNF-*α* induced apoptosis and improve behavioral defects in a mouse model of Parkinson's disease [29, 30].

Since CH has been used to treat forgetfulness for many years and its compounds have some effects on CNS, it maybe also has the neuroprotective effects on AD. In our study, we analyzed patients' cognitive and behavioral capacities and evaluated the effects on T-tau, TNF-*α*, and IL-1*β*. Potential effects of CH against neurodegeneration were observed.

## 2. Materials and Methods

### 2.1. Patients

All the patients provided a written informed consent. This study was carried out in accordance with the Code of Ethics of the World Medical Association (Declaration of Helsinki) and was approved by the ethics committee of Zhengzhou University (date: November 10, 2012).

26 participants were chosen from the second affiliated hospital of Zhengzhou University and the fifth affiliated hospital of Zhengzhou University from 2013 to 2014. They had similar education background and the mid-value length of symptoms before diagnosis was 1.5 years. Each participant included in our study had undergone a series of clinical interviews and examinations by the neurologist. Inclusion criteria were MMSE and ADAS-cog, from 10 to 20 and 29 to 40. Exclusion criteria were acute or chronic infection, original inflammatory disease, oncogenesis, therapy of Donepezil, memantine hydrochloride or Ginkgo biloba leaf, therapy of anti-inflammatory drug or corticosteroids, and C-reactive protein (CRP) level ⩾ 10 mg/L.

A total of 20 AD patients were constituted in drug therapy: CH treatment group: oral administration of Cistanches Herba capsule 48 weeks (0.9 g/day, each person, *n* = 11) and DON treatment group: oral administration of Donepezil tablet 48 weeks (5 mg/day, each person, *n* = 9). Six patients were enrolled as the control group (no treatment, *n* = 6). All participants agreed and were informed to take Cistanches Herba (CH) capsule (0.3 g/capsule, 3 capsules per day, LYYLYD pharmaceuticals Company, Changchun, China), Donepezil tablet (5 mg/tablet, 1 tablet per day, Eisai China Inc., Suzhou, China), or nothing, respectively. All patients had no any drug therapies before this study.

### 2.2. Cognitive Abilities Tests

All of the assessments were given before and after the treatments by blinded assessors.

#### 2.2.1. Minimental State Examination (MMSE)

MMSE was designed to evaluate the cognitive function of participant. It was composed of 30-point questionnaire tests in the scores that showed the changes in cognitive state. The range of MMSE score was from 0 to 30, and lower scores indicated greater severity of disease [31].

#### 2.2.2. Alzheimer's Disease Assessment Scale-Cognitive Subscale (ADAS-cog)

ADAS-cog was used to evaluate cognitive impairment in AD patients, the range of total scores was from 0 to 90, and higher scores indicated more serious of the disease [32].

Each patient had received a standardized medical evaluation that included MMSE and ADAS-cog before the medical treatment, at the time point after 24 weeks treatment and the time point after 48 weeks treatment. All data were computed according to criterion scoring instructions.

Mean scores were calculated and one-way ANOVA test was used to analyze differences among groups.

### 2.3. MRI

For each participant, MRI scans were performed on a GE 1.5 T Signa HDxt MRI scanner (General Electric Company, Fairfield, CT, USA). The MRI sequence was under the condition: TR = 11.6 ms, TE = 5 ms, spatial resolution = 0.94 mm, and slice thickness = 1.2 mm. All images were analyzed by using the software packages Analyze (Mayo Foundation, 1999) and Statistical Parametric Mapping (SPM99, Institute of Neurology, London, UK, exclusively used to measure volume of total gray matter) [33]. Hippocampal morphometry was managed by neuroradiologists (hand tracing) who were blind to diagnosis. Hippocampal outlines were drawn in the arrow-headed view using the “region of interest” tool implemented in the software Analyze. After having finished the drawing for all arrow-headed slices where the hippocampus was visible, the correctness of the horizontal was administrated in the coronal and sagittal views. Then, bilateral hippocampal volumes and the total brain volumes were determined using an automatic algorithm programmed in MATLAB and SPM99.

### 2.4. Lumbar Puncture

We collected the cerebrospinal fluid of patients at three time points: before the drug treatment, 24 weeks after treatment, and 48 weeks after treatment. None of the participants had lumbar puncture contraindications. Before performing the puncture, everyone should be told of the procedure along with potential risks and benefits and showed the consent from his or her guardian. The patient was in lying position with the back flexed. Next, the puncture point which was located in the intersection between the superior aspects of the iliac crest and the spine (L4 spinous level) was found. The landmark of the interspace between L3 and L4 space level was labeled. This location was below the termination of the spinal cord and much safer for this operation. Local anesthetic was infiltrated subcutaneously and spinal needle at the anatomical landmark was injected along the intended path. Eight milliliters fluid were collected and stored at −80°C for further test. When an adequate specimen was obtained, needle was removed and the patient lied flat for another 6 hours.

### 2.5. Reverse Transcription-Polymerase Chain Reaction (RT-PCR)

We used the Trizol reagent (CWBIO, Beijing, China) to extract total RNA from the CSF of the participants according to the manufacturer's instruction and generated first-strand cDNA by ThermoScript RT-PCR System (Invitrogen, Grand Island, New York, USA) with 1 *μ*L random hexamer primer, 2 *μ*L 10 mM dNTP-Mix, 0.1 M DTT, and 1 *μ*L ThermoScript RT in DEPC-treated water to a 20 *μ*L final reaction volume. Then reaction mixture denatured it at 63°C for 5 minutes, followed at 45°C at 50 minutes, and stored at −20°C.

According to the data of gene bank, we designed the primers and synthesized them by Sangon Biotech (Shanghai, China): T-tau, forward: 5′-CAAAGCTCGCATGGTCAGT A-3′ and reverse: 5′-AGGGTTGGATCAGAGGGTCT-3′; TNF-*α*, forward: 5′-AGGC AATAGGTTTTGAGGGCCAT-3′ and reverse: 5′-GGGACACACAAGCATCAAGGA TAC-3′; IL-1*β*, forward: 5′-TGGCAGGTTCCTGATTT-3′ and reverse: 5′-AACTTGTT CGACAA CCCTC-3′; control GADPH, forward: 5′-GGUCAUCCAUGACAAC UUUTT-3′ and reverse: 5′-AAAGUUGUCAUGGAUGACCTT-3′. These expression levels of mRNA were measured with RT-PCR. The reaction mixture consisted of 1 *μ*g of sample cDNA, 1 *μ*L primer, 2 *μ*L of 10 mM dNTPs, 25 *μ*L Taq DNA polymerase (Sangon Biotech), and DEPC-Treated water to final volume of 50 *μ*L. The PCR conditions were 98°C for 30 seconds (denaturation), 55–58°C for 30 seconds (annealing), and 72°C for 60 seconds (extension) for 30 cycles and then at 72°C for 10 minutes, stored it at 4°C. The fragments obtained were confirmed by 1.0% agarose gel electrophoresis. The PCR products sizes of T-tau, TNF-*α*, and IL-1*β* were 188 bp, 560 bp, and 298 bp.

### 2.6. Western Blot

Protein preparation for Western blot assay [34] is as follows: 1 mL sample of CSF sample added with 250 *μ*L of 50% trichloroacetic acid (Sigma-Aldrich, St. Louis, Missouri, USA) was centrifuged at 17,000 ×g at 4°C for 20 minutes; After supernatents were discarded, the pellet was washed with ethyl ether (Merck Co, Kenilworth, New Jersey, USA), and the dissolved protein pellet was in a solution including 4% CHAPS, 40 mmol/L Tris base, 8 mol/L urea, and 100 mmol/L dithiothreitol. After the precipitation of CSF samples, 50 *μ*g of the protein lysate mixed with buffer was denatured at 70°C for 10 minutes. Then, the protein was electrophoretically separated on 12% SDS-PAGE gels and transferred onto an Immobilon-P polyvinylidene fluoride (PVDF) membranes (Amersham Biosciences, Bath, UK). The membranes were blocked in 5% nonfat milk in Tris-Buffered Saline (TBS) for 1 hour at room temperature (RT) and then incubated with primary antibody in TBS containing 0.1% Tween-20 overnight at 4°C. After the membrane was washed three times with TBST, it was incubated with the secondary antibody for 1 hour at RT. Human monoclonal *β*-actin antibody (1 : 5000, Sigma-Aldrich) was used as a loading control. An enhanced chemiluminescence system (Sigma-Aldrich) was used for the visualisation and quantification. Imager Gel Doc System and Quantity One Software (Bio-Rad, Richmond, California, USA) were used to analyze the densitometric intensities.

### 2.7. Enzyme Linked Immunosorbent Assay (ELISA)

Concentrations of T-tau were measured by sandwich ELISA (Innotest hTAU-Ag; Innogenetics, Ghent, Belgium). TNF-*α* and IL-1*β* were tested by ELISA using human cytokine/chemokine Luminex kits (for TNF-*α* and IL-1*β*) from Millipore (St-Quentin-en-Yvelines, Yvelines, France). All experiments were performed as recommended according to the manufacturer's instructions. The 96-well plates were prewet and incubated in with 200 *μ*L of assay buffer for 10 minutes at 37°C. Samples were added in duplicate to the suitable wells. And assay buffer was used as blank control. Plates which were loaded with magnetic microbead solution priorly were sealed and incubated with agitation on a plate shaker at 750 rpm at 4°C in darkroom. 200 *μ*L of wash buffer was used to wash each well twice and 25 *μ*L of detection antibodies was added per well. Plate was incubated on a plate shaker at 750 rpm for 1 h at 37°C in darkroom and then washed twice by 200 *μ*L of wash buffer again. Microbeads were resuspended in sheath fluid 100 *μ*L per well of on plate shaker at 450 rpm for 10 minutes at 37°C. Values were read at 450 nm on a microplate reader (BioRad).

### 2.8. Statistical Analysis

All experiments data were analyzed by SPSS 13.0 software. Quantitative data were presented as mean ± SD. One-way ANOVA test was used to analyze differences in cognitive abilities, volume of hippocampus, T-tau, TNF-*α* and IL-1*β* mRNA levels, and protein expressions and concentrations obtained by ELISA among groups. Bonferroni was used between the two-group comparison. The least significant difference (LSD) was used to compare difference between the two groups. *P* < 0.05 was considered statistically significant.

## 3. Results

### 3.1. Cistanches Herba Could Improve Cognitive Abilities

**Figure 1:**
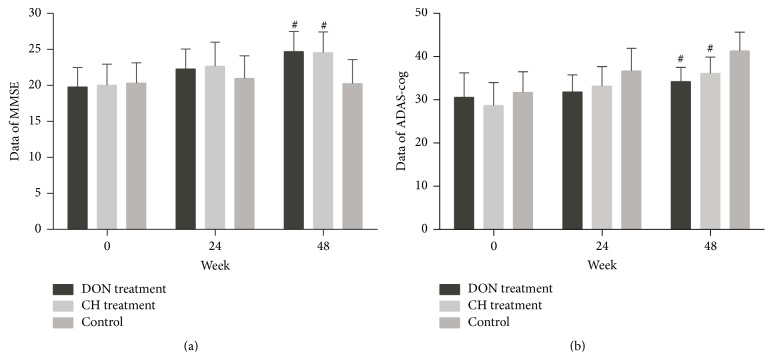
Participant outcomes at pre-and posttreatment. Data were expressed as mean ± SD. (a) MMSE: minimental state examination; (b) ADAS-cog: Alzheimer's disease assessment scale-cognitive subscale; outcomes of drug treatment groups were better than control group at 48 weeks (^#^ *P* < 0.05).

10 of the 11 participants in the CH group (the retention rate was 90.1% and the attrition rate was 9.9% and one patient quitted this study because of serious vomiting), 8 of the 9 participants in the Don group (the retention rate was 88.9% and the attrition rate was 11.1% and one patient quitted because of peptic ulcer), and 6 of the 6 participants in the control group (the retention rate was 100%) completed the 48 weeks open-label pilot study. The clinical characteristics of AD patients were presented in Table 1. AD participants with drug treatments or without therapies had their cognitive abilities evaluated by MMSE and ADAS-cog, at the follow-up period (Figures 1(a) and 1(b)). The measure in drug treatment groups showed higher scores using MMSE and lower scores using ADAS-cog at 48 weeks (*P* < 0.05), compared with control group. However, there were no obvious differences among groups at 24 weeks, and the data showed little difference between CH and DON groups (*P* > 0.05).

### 3.2. Cistanches Herba Could Slow Down Hippocampus Atrophy

**Figure 2:**
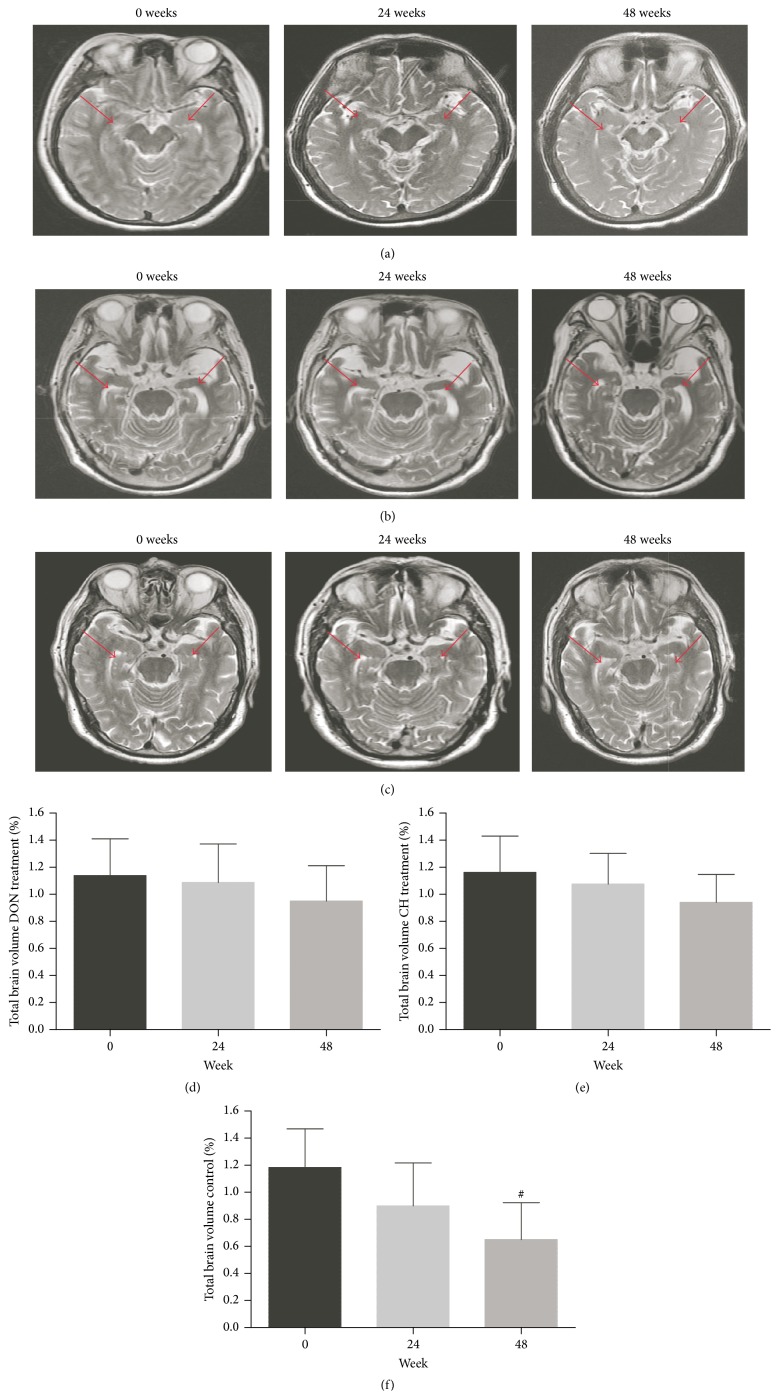
Ratios of bilateral hippocampus volume measured by MRI. For (a)–(c) MRI images of DON treatment group (a), CH treatment group (b), and control group (c). The right side of the brain was on the right side of the image. For (d)–(f): decrease of the volume of bilateral hippocampus (HIP) was remarkable in control group between the mean of data measured at 48 weeks and at 0 weeks (^#^ *P* < 0.05). There were no visible volume changes of hippocampus in drug treatment groups (*P* > 0.05). Volume was accounted as a percentage of the total brain volume for each patient.

The volume of bilateral hippocampus (HIP) in control group measured at 48 weeks was 4.2% below the mean of data measured at 0 weeks. And there were no visible volume changes of hippocampus in drug treatment groups (Figures 2(a)–2(f)).

### 3.3. Cistanches Herba Could Reduce the Expression of T-tau, TNF-*α*, and IL-1*β* mRNAs and the Proteins Levels of T-tau, TNF-*α*, and IL-1*β*

**Figure 3:**
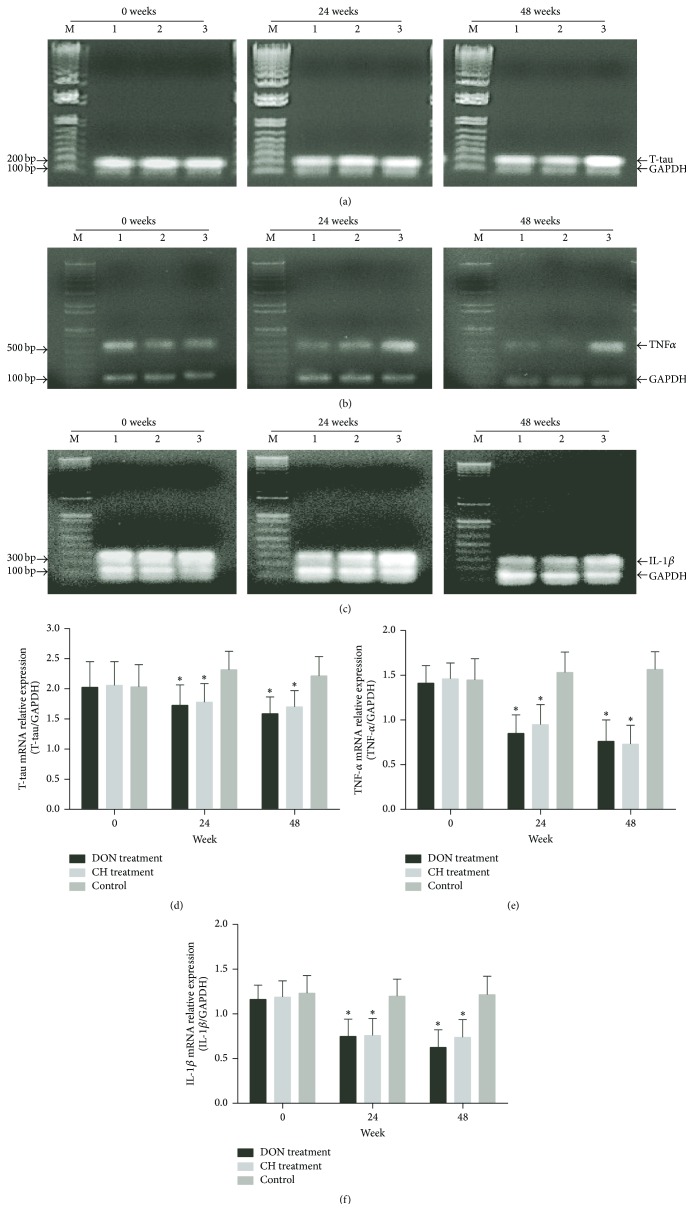
T-tau, TNF-*α*, and IL-1*β* mRNA expressions in the CSF of AD patients evaluated by RT-PCR. The mRNA expressions of T-tau ((a), 188 bp), TNF-*α* ((b), 560 bp), and IL-1*β* ((c), 298 bp) from AD patients' CSF, GAPDH (100 bp). 1: DON treatment group; 2: CH treatment group; 3: control group. For (d)–(f): the relative changes in mRNA expressions of T-tau, TNF-*α*, and IL-1*β*. The mRNA expressions of T-tau, TNF-*α*, and IL-1*β* had reduced during 48 weeks compared with control group after drug treatments (^*∗*^ *P* < 0.01). There was no statistical deviation between DON treatment group and CH treatment group (*P* > 0.05). M: marker.

**Figure 4:**
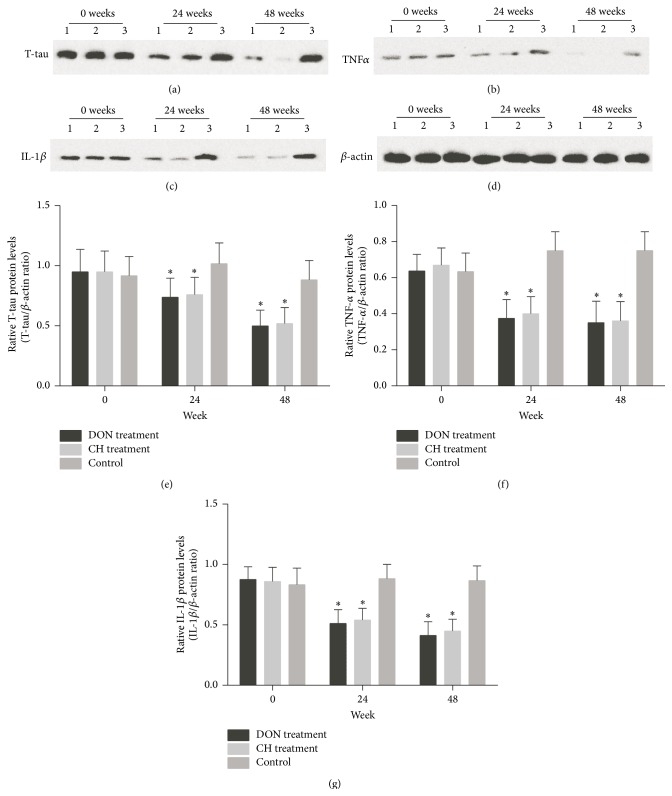
T-tau, TNF-*α*, and IL-1*β* protein levels in the CSF of AD patients measured by Western blot. Protein levels of T-tau (a), TNF-*α* (b) and IL-1*β* (c) from AD patients' CSF, and (d) *β*-actin. 1: DON treatment group; 2: CH treatment group; 3: control group. For (e)–(g) the relative changes in protein content of T-tau, TNF-*α*, and IL-1*β*. The contents of T-tau, TNF-*α*, and IL-1*β* had decreased during 48 weeks compared with control group after drug treatments (^*∗*^ *P* < 0.01). There was no statistical deviation between DON treatment group and CH treatment group (*P* > 0.05).

T-tau, TNF-*α*, and IL-1*β* mRNAs were downregulated significantly in CH treatment group and DON treatment group, respectively (Figures 3(a)–3(c)) during 48 weeks. The levels of T-tau, TNF-*α*, and IL-1*β* mRNAs showed a diminishing tendency (Figures 3(d)–3(f)). T-tau, TNF-*α*, and IL-1*β* proteins also declined in CH treatment group and DON treatment group compared to control (Figures 4(a)–4(g)). No differences were found in either expressions of mRNAs or protein levels between CH treatment group and DON treatment group, indicating Cistanches Herba could reduce T-tau, TNF-*α*, and IL-1*β* mRNAs and protein levels as the same effects as Donepezil did.

### 3.4. Cistanches Herba Could Inhibit the Secretions of T-tau, TNF-*α*, and IL-1*β*

**Figure 5:**
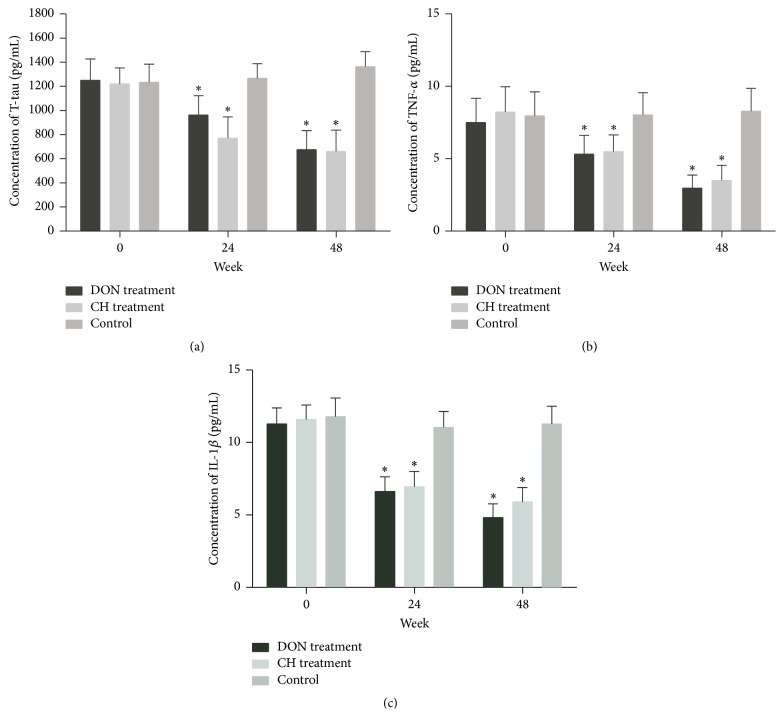
Changes of T-tau, TNF-*α*, and IL-1*β* in CSF of AD patients determined by ELISA assay. (a) T-tau. (b) TNF-*α*. (c) IL-1*β*. T-tau, TNF-*α*, and IL-1*β* concentrations had attenuated during 48 weeks compared with control group after drug treatments (^*∗*^ *P* < 0.01). No statistical difference between DON treatment group and CH treatment group (*P* > 0.05).

Cistanches Herba was reported to be involved in many types of inflammatory diseases [35]. We measured the contents of TNF-*α* and IL-1*β* in CSF by ELISA. Meanwhile, we assessed concentrations of T-tau in CSF by ELISA. Compared with the control group, T-tau, TNF-*α*, and IL-1*β* levels in CSF were lower (*P* < 0.01) in treatment groups, as shown in Figures 5(a)–5(c), indicating that Cistanches Herba could reduce T-tau levels and inhibit inflammatory factor such as TNF-*α* and IL-1*β* secretions in AD pathologic process. Between two treatment groups, T-tau, TNF-*α*, or IL-1*β* contents had no statistical difference (*P* > 0.05), suggesting that both drugs could reduce T-tau, TNF-*α*, and IL-1*β* levels and the effect of Cistanches Herba was the same as Donepezil. Compared with the control group, T-tau, TNF-*α*, and IL-1*β* contents in the treatment groups had a decreasing tendency.

## 4. Discussion

Our studies showed that Cistanches Herba could improve cognitive abilities, slow down hippocampus atrophy, and reduce the contents of T-tau, TNF-*α*, and IL-1*β* in the CSF of moderate AD patients. We analyzed relative hippocampal volumes performed by the quotient of hippocampal and total brain volume and made the correctness of the horizontal by being controlled in the coronal and sagittal views. It was maximumly able to reduce errors. No previous researchers had reported the neuroprotective effects of Cistanches Herba on AD patients. To the best of our knowledge, this study is the first to explore the therapeutic effects of Cistanches Herba in AD patients.

“Yin/Yang Theory” is a classic structure of Traditional Chinese medicine (TCM), which believes the human body structure is an organic entity and the universe is a unity of opposing strength, that is, Yin and Yang. According to Yin/Yang Theory, Qi is classified to Yang, while the essence, blood, and body fluids belong to Yin. All of them build the basis of the human body. With the Yin/Yang concept, TCM explains clinical symptoms and guides treatment of diseases. Yang/Qi drives the biological activities in the human body and enhances energy in human organs. Shortages in Yang/Qi display an important role in Alzheimer's disease [36]. Cistanches Herba is thought as a material of Yang-invigoration in human body. It may be a treatment choice to AD. In our study, we tried to observe the effects of Cistanches Herba on AD by the theory of Western medicine. Meanwhile, we choose Donepezil to compare with Cistanches Herba to evaluate its medicine effects. Donepezil, a well-known acetylcholinesterase inhibitor (AChE) approved by FDA, was proved to protect from the A*β* and tau toxicity in animal models and cell [37]. The 1-year, placebo-controlled trial had revealed that donepezil-treated patients attenuated by about half as much as the placebo-treated patients on a global rating of dementia symptoms [38]. And the prior research demonstrated that an administration of donepezil to AD patients for 1 month led to decreased levels of IL-6 and IL-1 in mononuclear cells of peripheral blood, indicating the anti-inflammatory influence of this agent. Donepezil also had proved the anti-inflammatory effects in apolipoprotein E-knockout mice that showed restrained expression of TNF-*α* in the aorta [39].

We studied the influence of Cistanches Herba in order to investigate whether it could play a key role in the pathophysiology of AD in CSF. A significant statistical difference of cognitive abilities between drug treatment groups and control group was observed. Mohs et al. [40] also reported that Donepezil benefits in enhancing cognitive abilities of AD. The analysis of T-tau, TNF-*α*, and IL-1*β* expressions in CSF we performed showed that all of them changed significantly during 48 weeks by the therapies. Our findings showed the mRNA expressions of T-tau, TNF-*α*, and IL-1*β* and the levels of these proteins reduced after the therapy by Cistanches Herba, and there were decreasing tendencies with the prolongation of treatment time. It suggested Cistanches Herba could cross blood-brain barrier (BBB) and involved the pathological process of AD. Cistanches Herba may be able to restore a normal brain regulatory network.

Recent studies reported that carbohydrates in Cistanches Herba had central nervous system (CNS) effects and it could inhibit inflammatory cytokines activities [24, 41]. It may be due to some kinds of active ingredients. Phenylethanoid glycosides (PhGs), separated and purified from Cistanches Herba, are regarded as the primary bioactive elements, which many studies have shown that they have broad biological effects involving antibacterial, cytotoxic, anti-inflammatory, and neuroprotective activities. In Mannami's study, PhGs were able to inhibit the aggregation of A*β*42 and restrain A*β*42-induced cytotoxic effects on human neuroblastoma SH-SY5Y cells by stimulating the expression of mRNA of glycolytic enzymes and intracellular ATP [42]. PhGs could increase the spatial learning memory of apolexis accelerated-prone mice by enhancing the mRNA expression of phosphoglycerate kinase [43]. Park's trails showed a 17 kD N-terminal tau fragment generated by calpain cleavage, containing residues amino acid 45–230, which was arranged to mediate A*β*-induced toxicity [44]. The researches on tau immunotherapy suggest that tau immunization produces the activation of complement system that induces a bystander effect that leads to proteolysis of A*β* [45]. Those mean some relationship between A*β* and tau. However, little relative researches lied in discussion about the interactive effects of A*β* and tau or PhGs and tau protein. In our studies we speculated PhGs might affect the tau pathology in AD by influencing aggregation of A*β*, but the clear mechanism was unknown. The researches on anti-inflammatory capabilities in vitro reported that PhGs could inhibit IL-10 and TNF-*α* production in peritoneal macrophages. They also were proven to have dose-dependent effects on inhibitions of both gene and protein expressions of chemokines activated by interferon- (IFN-) g and TNF-*α* [46]. However, in our study we did not isolate PhGs from Cistanches Herba and focus on them. Further researches with different approaches such as cell biology, molecular biology, and plant chemistry would be needed to picture the actions of Cistanches Herba and its components.

Inflammation is a known factor in the etiopathogenesis of neurodegenerative diseases, such as Alzheimer's disease (AD) [47]. Early studies have proved that the native immune system and the complement system in brains of AD patients are activated. And the amyloid plaques are always surrounded by activated microglia, which can active nod-like receptors (NLRs) in the cytoplasm. These NLRs lead to the activation and concentration of cytosolic protein complexes as inflammasomes. These complexes can activate proinflammatory caspases, specifically caspase-1, which then makes the proinflammatory cytokines such as TNF-*α* and IL-1*β* activated [48]. The previous study reported that Cistanches Herba improved the immune system [49, 50]. Our studies demonstrated that the levels of TNF-*α* and IL-1*β* in CFS of drug treatment groups were significant lower than that in control group on 24 weeks and 48 weeks. We hypothesized that Cistanches Herba might be connected with the neuroinflammation pathway in AD brains.

## 5. Conclusions

Our study demonstrated Cistanches Herba could improve cognitive abilities in AD and slow down patient's hippocampus atrophy, suppress the expressions of T-tau, TNF-*α*, and IL-1*β* in CSF of AD patients as the similar efficacy as Donepezil, but Cistanches Herba was much cheaper and easier to get, so it might be a kind of accessible substitute for Donepezil, especially in China or other developing countries.

## Figures and Tables

**Table 1:** Clinical characteristics of the moderate Alzheimer disease patients.

	DON group (*n* = 8)	CH group (*n* = 10)	control (*n* = 6)
Gender (%women)	62.5	60	50
Education (years)	14.6 ± 3.9	15.5 ± 3.4	15.7 ± 2.9
Age (years)	73.5 ± 5.0	70.3 ± 6.4	71.3 ± 5.4

## Abbreviations

CH: Cistanches Herba
DON: Donepezil
PhGs: Phenylethanoid glycosides
TCM: Traditional Chinese medicine.
